# Editorial: *Streptococcus* spp. and *Corynebacterium* spp.: Clinical and Zoonotic Epidemiology, Virulence Potential, Antimicrobial Resistance, and Genomic Trends and Approaches

**DOI:** 10.3389/fmicb.2022.867210

**Published:** 2022-02-28

**Authors:** Prescilla Emy Nagao, Andreas Burkovski, Ana Luíza Mattos-Guaraldi

**Affiliations:** ^1^Laboratory of Molecular Biology and Physiology of Streptococci, Institute of Biology Roberto Alcantara Gomes, Rio de Janeiro State University – UERJ, Rio de Janeiro, Brazil; ^2^Microbiology Division, Department of Biology, Friedrich-Alexander-Universität Erlangen-Nürnberg, Erlangen, Germany; ^3^Laboratory of Diphtheria and Corynebacteria of Clinical Relevance, Faculty of Medical Sciences, Rio de Janeiro State University – UERJ, Rio de Janeiro, Brazil

**Keywords:** *Streptococcus* spp., *Corynebacterium* spp., epidemiology, virulence, antimicrobial resistance, genomic features

*Streptococcus* genus comprises pathogens implicated in human and animal diseases, economic losses to agriculture, in addition to species employed in dairy industry. Molecular genetics, taxonomic approaches, and phylogenomic investigations detected more than 100 *Streptococcus* spp. and more than nine subspecies. Studies also contributed to the understanding of highly complex features of pathogenic potential and multiple virulence mechanisms (Lannes-Costa et al., 2021), in addition to antimicrobial resistance with impact on health systems worldwide (Haenni et al., 2018).

Nowadays, *Corynebacterium* has more than 132 species, including at least 50 species of medical, veterinary, and/or biotechnological relevance. Despite global immunization programs, diphtheria outbreaks and atypical cases of diphtheria, localized, and/or systemic infections independent of diphtheria toxin production by *Corynebacterium diphtheriae* and *Corynebacterium ulcerans* zoonotic pathogen, still occur in industrialized and developing countries. Cases of infections related to different and new non-diphtherial *Corynebacterium* species have been favored by genotyping, virulence, taxonomy studies, and/or laboratorial identification techniques (Zasada and Mosiej, 2018; Silva-Santana et al., 2021).

*Streptococcus* spp. and non-diphtherial *Corynebacterium* species expressing multidrug-resistance profiles (MDR) have been reported with increased frequency as pathogens of invasive infections and/or nosocomial outbreaks. A detailed understanding of multifactorial pathogenic mechanisms is essential to develop new therapeutic approaches, surveillance and control strategies of streptococcal, diphtheria, and non-diphtherial corynebacterial diseases, including adhesive activities, biofilm formation, metabolite exchange, cellular communication, protection to antimicrobials, and against host immune attacks. Therefore, additional studies remain necessary to investigate phenotypic and genotypic properties of virulence mechanisms and resistance to antimicrobial agents involved in multifactorial and complex adaptation strategies to host environmental conditions. Invasive medical devices and/or empirical antibiotic therapy may contribute to dissemination of invasive infection in hospitalized patients (Ramos et al., 2019; Figueiredo et al., 2021; Henares et al., 2021).

We thank all the authors who provided relevant aspects of *Streptococcus* spp. and *Corynebacterium* spp. to this Research Topic by analyzing different features concerning virulence, multidrug resistance, biofilm production, potential target, genetic, and environmental factors.

*Streptococcus pneumoniae* is an important human pathogen that can cause severe invasive pneumococcal diseases (IPDs). In a multicenter study of Zhou et al. investigated serotypes, sequence type distribution, antimicrobial susceptibility, and virulence of *S. pneumoniae* invasive strains causing IPD in China. Data provided insight into the epidemiology and virulence diversity of *S. pneumoniae* strains, including capsular polysaccharide and non-capsular virulence factors. The isolation rate of serogroup 15 *S. pneumoniae* has been increasing since developing countries began administering the 13-valent pneumococcal conjugate vaccine. Shi et al. verified that serogroup 15 *S. pneumoniae* presenting 78.57% multidrug resistance rate is common among children in China, and consequently, these strains should be continuously monitored.

*Streptococcus agalactiae* (group B *Streptococcus*) is one of the most important agent of bovine mastitis and causes remarkable direct and indirect economic losses to the livestock sector. Moreover, this species can cause severe human diseases in susceptible individuals. The study conducted by Carra et al. showed that human and bovine isolates strains shared the same antibiotic resistance profiles supporting the hypothesis of interspecies transmission of *S. agalactiae* between bovines and humans.

*Streptococcus pyogenes* (group A *Streptococcus*; GAS) is an important pathogen for humans often associated with severe and invasive diseases. No vaccine exists, so antibiotics are essential for effective treatment. Even though this pathogen remains universally susceptible to penicillin, therapeutic failures have been reported in some GAS illnesses. Additional studies remain necessary to fully explain and elucidate the mechanisms of antibiotic-unresponsive infections. Martini et al. showed that some GAS strains could form antimicrobial persisters during biofilm formation on abiotic surfaces. Gene expression assays showed upregulation of some genes associated with efflux pumps in persisters strains arising in the presence of penicillin. This event was due to non-inherited resistance mechanisms. Multifactorial mechanisms involving protein synthesis inhibition, cell growth impairment, and efflux pumps seemed to play roles in the formation of antimicrobial persisters in *S. pyogenes*. In their review, Johnson and LaRock discussed the challenges of treating GAS infection, mechanisms that contribute to antibiotic failure, and adjunctive therapeutics for improving the treatment of high-risk GAS infections by non-susceptible or resistant isolates.

*Streptococcus mutans* is considered the prime pathogen of dental caries. Li et al. provided new insights into the thoroughly investigated mechanism of microbial fluoride tolerance, and suggested F0F1-ATPase as a potential target for suppressing fluoride resistant strains. Moreover, *S. mutans* can secrete glucosyltransferases (GTFs) to synthesize extracellular polysaccharides, which are the virulence determinants of cariogenic biofilms. Ursolic acid, a type of pentacyclic tri terpene natural compound, was verified to decrease bacterial viability and prevent *S. mutans* biofilm formation by binding and inhibiting the activity of GTFs (Liu et al.).

In recent years, reports of infections and nosocomial outbreaks caused by antimicrobial multidrug-resistant *Corynebacterium striatum* strains have been increasing worldwide. Despite the different existing mobile genomic elements, there is evidence that acquired resistance genes are coupled to insertion sequences in *C. striatum*. This perspective article reviewed the insertion sequences linked to resistance genes, their relationship to evolutionary lineages, epidemiological characteristics, and the niches the strains inhabit. The potential of the insertion sequences for their application as a descriptor of epidemiological scenarios, allowing us to anticipate the emergence of multidrug-resistant lineages was also discussed (Leyton-Carcaman and Abanto). Dover et al. reported phylogenomic reappraisal of fatty acid biosynthesis, mycolic acid biosynthesis and clinical relevance among members of the genus *Corynebacterium*. Data suggested that although a mycolic acid-based mycomembrane is widely considered the target for interventions by the immune system and chemotherapeutics, the structure is not essential in corynebacteria and is not a prerequisite for pathogenicity or colonization of animal hosts. In the investigation using a mouse model for *Corynebacterium* in which colonization with either *Corynebacterium accolens* or *Corynebacterium amycolatum*, significantly reduced *S. pneumoniae* acquisition in the upper airway and infection in the lung. The lungs of co-infected mice had reduced pro-inflammatory cytokines and inflammatory myeloid cells, indicating resolution of infection-associated inflammation. Lipase-dependent and independent effects, indicating that both this and other bacterial factors contribute to inhibitory effects of *C. accolens* and *C. amycolatum* on *S. pneumoniae* (Horn et al.).

Finally, we would like to thank the people that supported this Research Topic and the reviewers for their time and comments that helped to improve the manuscripts.

## Author Contributions

PN, AB, and AM-G wrote and edited the manuscript. All authors have made substantial contributions to the article and approved the manuscript for publication.

## Funding

The authors and their work were supported by Fundação de Amparo à Pesquisa do Estado do Rio de Janeiro (FAPERJ), Conselho Nacional de Desenvolvimento Científico e Tecnológico (CNPq, Brazil), Sub Reitoria de Pós-Graduação e Pesquisa da Universidade do Estado do Rio de Janeiro (SR-2/UERJ), and Coordenação de Aperfeiçoamento de Pessoal de Nível Superior–Brasil (CAPES)–Finance Code 001.

## Conflict of Interest

The authors declare that the research was conducted in the absence of any commercial or financial relationships that could be construed as a potential conflict of interest.

## Publisher's Note

All claims expressed in this article are solely those of the authors and do not necessarily represent those of their affiliated organizations, or those of the publisher, the editors and the reviewers. Any product that may be evaluated in this article, or claim that may be made by its manufacturer, is not guaranteed or endorsed by the publisher.
